# Concentration and Potential Ecological Risk of PAHs in Different Layers of Soil in the Petroleum-Contaminated Areas of the Loess Plateau, China

**DOI:** 10.3390/ijerph15081785

**Published:** 2018-08-20

**Authors:** Di Wang, Jing Ma, Hao Li, Xingchang Zhang

**Affiliations:** 1 College of Natural Resources and Environment, Northwest A&F University, Yangling 712100, China; wd4591@126.com; 2 Inspection and Testing Center for Quality and Safety of Agricultural Products, Ningxia Institute of Agricultural Survey and Design, Yinchuan 750002, China; yajing2345678@163.com (J.M.); cpu_rs@163.com (H.L.); 3 State Key Laboratory of Soil Erosion and Dryland Farming on the Loess Plateau, Institute of Soil and Water Conservation, Northwest A&F University, Yangling 712100, China

**Keywords:** polycyclic aromatic hydrocarbons (PAHs), petroleum-contaminated, concentration, ecological risk, Loess Plateau

## Abstract

The three most representative areas of petroleum pollution on the Loess Plateau are the research subjects of this study. In this study, 16 priority polycyclic aromatic hydrocarbons (PAHs) were determined by the QuEChERS method combined with gas chromatography-tandem mass spectrometry (GC-MS/MS). The total concentrations of ∑16PAHs in top layer soils (0–10 cm), middle layer soils (10–30 cm), and bottom layer soils (30–50 cm) ranged from 1010.67 to 18,068.80, 495.85 to 9868.56 and 213.16 to 12,552.53 μg/kg, with an average of 5502.44, 2296.94 and 2203.88 μg/kg, respectively. The 3-ring and 4-ring PAHs were the most prominent components in all soil samples. Meanwhile, the average value of ∑16PAHs decreased with the depth, from 5502.44 μg/kg (0–10 cm) to 2203.88 μg/kg (30–50 cm). The PAHs levels in the studied soils were heavily polluted (over 1000 μg/kg) according to the Soils Quality Guidelines and 95% of PAHs come from petroleum sources. Moreover, the total of PAHs in petroleum-contaminated soils was assigned a high ecological risk level. Toxic equivalency quantities (TEQs) indicated that PAHs in petroleum-contaminated soils presented relatively high toxicity.

## 1. Introduction

Polycyclic aromatic hydrocarbons (PAHs) are a class of diverse organic compounds containing two or more fused aromatic rings made up of carbon and hydrogen atoms [1]. Generally, they are produced from incomplete combustion of organic materials, fossil fuels, petroleum product spillage and various domestic and industrial activities [2,3]. Once emitted, PAHs can be widely dispersed in air, water, soil and sediment. Due to the hydrophobicity and lipophilicity of PAHs, soil is the most important sink for PAHs in natural environment [4,5]. It has been reported that soil can store approximately 90% of PAHs [6]. PAHs in soils can be carried into surface/ground water through precipitation and surface runoff, emitted into atmosphere by volatilization, and transported into crops from polluted soil and air via root and leaf adsorption, which may further accumulate in human and other organisms via food chains [7]. Thus, monitoring the concentration of PAHs in soils is important for understanding its environmental fate.

Many PAHs are mutagenic and some are carcinogenic, raising concerns over their occurrence in the environment [8,9]. Based on their potential toxicity, the United States Environmental Protection Agency (USEPA) has identified 16 PAHs as priority pollutants [10]. Meanwhile, the USEPA and the International Agency for Research on Cancer (IARC) have also considered 7 of 16 priority PAHs as probable/possible human carcinogens. In addition, they are considered as candidates of persistent organic pollutants (POPs) that merit further investigation for possible early listing in the Stockholm Convention on POPs. Thus, more attention has been paid to PAHs in recent years [11,12,13]. However, there has been less research on the concentration, distribution and possible sources of PAHs in petroleum-contaminated soils, as compared to urban and agricultural soils.

In recent years, China has arduously implemented the One Belt One Road initiative, and constructed what is known as the New Silk Road. Because the countries and terrains along this route are rich in oil and gas resources, it is expected to very soon become an ‘Energy Road’. The Loess Plateau is one such main terrain area along the Silk Road Economic Belt, and it is also the key energy base in China. The Loess Plateau has abundant oil and gas resources. The most abundant oil resources on the Loess Plateau are specifically distributed in Yulin, Yan’an and Qingyang. With the large-scale exploitation of these petroleum resources, the ecological environment has become severely polluted. Even though this region has large geological reserves, with wide distribution petroleum-rich areas, yet the peculiar geographical structures limit the reservoir scale within a relatively small area. Oil wells are plentiful, yet not well connected. This makes it very difficult to systematically monitor petroleum contamination. Consequently, our research on petroleum-contaminated soils addresses an urgent need.

Furthermore, petroleum is a complex mixture of alkanes, aromatics, resins, asphaltenes, and other organic matter [14]. Of all petroleum components, PAHs are considered the most important. Petroleum and its derivatives are easily released into the environment during petroleum extraction, storage and transportation. These processes not only entail wastage of precious petroleum resources, but also pollute and destroy the ecological environment, and endanger human health. Therefore, it is imperative to conduct research on petroleum-contaminated soils.

On the whole, these three representative areas (Yulin, Yan’an and Qingyang) with petroleum pollution on the Loess Plateau are the research subjects of this study. The main objective of the present study was to determine the concentration levels of PAHs in petroleum-contaminated soils, and to assess the probable sources of PAHs contamination. Additionally, the ecological risk and toxicity of PAHs in soils were evaluated. The results obtained may significantly provide basic theoretical data for the PAHs remediation.

## 2. Materials and Methods

### 2.1. Study Area Description

The Loess Plateau is located in the north-central part of China and is one of the four major highlands in China. It extends over 8 latitudes (34–41° N) and 14 longitudes (101–114° E), with a total area of 640,000 km^2^. It covers almost all of the provinces of Shaanxi and Shanxi and extends into parts of Gansu, Ningxia, and Inner Mongolia. It has a semi-arid climate, with extensive monsoonal influence. The average annual temperature ranges from 6 to 14 °C. The soil type is classified as typical loessal soil, which is easily eroded, causing nutrient deficiency. Yulin, Yan’an and Qingyang, as key research areas, are distributed from north to south on the Loess Plateau.

### 2.2. Sample Collection

**Figure 1:**
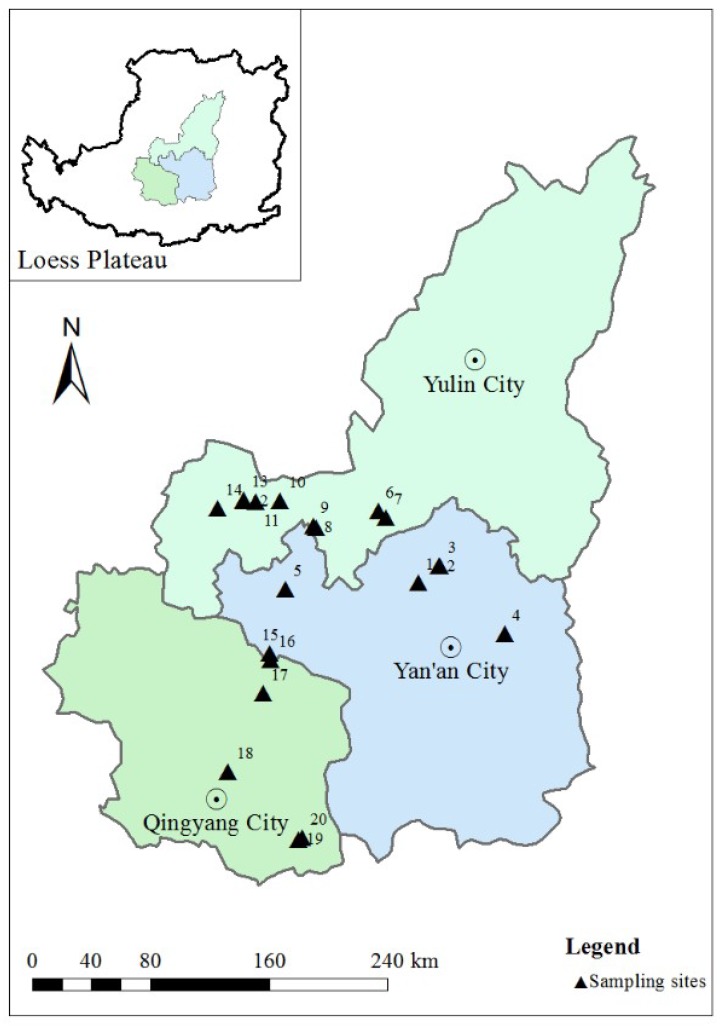
Location of the sampling sites on the Loess Plateau.

The sampling sites (35°28′44′′ N–37°30′41′′ N, 107°42′12′′ E–109°53′5′′ E) are located on the Loess Plateau. A total of 60 petroleum-contaminated soil samples were collected from 20 sampling sites in July 2017. Soil samples (0–10 cm depth, 10–30 cm depth and 30–50 cm depth) were taken with a stainless steel soil auger after removal of the uppermost cover. Five samples were gathered over an area of 100 m^2^, mixed to form a composite sample [15]. During the whole sampling process a global position system (GPS) was used to accurately provide the location of each sampling point as shown in Figure 1. The basic information of the sampling sites in details is given in Table 1. After transport to the laboratory, the soil samples were air dried, ground, passed through a 60-mesh screen, homogenized, and stored at 4 °C until analysis.

### 2.3. Reagents and Standards

A standard mixture containing 16 PAHs: naphthalene (NAP), acenaphthylene (ACY), acenaphthene (ACE), fluorene (FLU), phenanthrene (PHE), anthracene (ANT), fluoranthene (FLA), pyrene (PYR), benz(а)anthracene (BаA), chrysene (CHR), benzo(b)fluoranthene (BbF), benzo(k)fluoranthene (BkF), benzo(а)pyrsne (BаP), indeno(1,2,3-c,d)pyrene (InP), dibenz(а, h)anthracene (DBA), benzo(g,h,i)perylene (BgP), was purchased from AccuStandard Inc. (New Haven, CT, USA). High Performance Liquid Chromatography (HPLC) grade dichloromethane was purchased from Waters Company (Milford, MA, USA). The other reagents were all analytical grade. QuEChERS extraction kits containing 50 mg C18, 150 mg PSA and 900 mg Na_2_SO_4_ were provided by Agilent Technologies Inc. (Santa Clara, CA, USA). Milli-Q water was used to perform the analytical procedures.

### 2.4. Sample Extraction

In the laboratory, the samples were air-dried at room temperature and stones, roots and other debris were removed. The samples were then ground and sieved through a 60-mesh screen. Soil samples (2.0 g) were mixed with anhydrous sodium sulfate (3.0 g), and extracted with dichloromethane (20 mL) for 30 min under ultrasoound. After centrifuging the tubes at 9500 r/min for 10 min, a 2-mL supernatant sample was transferred to a single-use centrifuge tube containing 150 mg of PSA, 50 mg of C18, and 900 mg of anhydrous Na_2_SO_4_. The mixtures were shaken vigorously for 1 min using a vortex mixer to ensure that the solvent contacted the entire sample. Subsequently, the samples were centrifuged at approximately 9500 r/min for 10 min. Then, the upper layer of the prepared sample was filtered through a 0.22 μm syringe filter and transferred to an autosampler vial for injection.

### 2.5. Instrumental Analysis

The determination of PAHs was performed on GCMS-TQ8040 (Shimadzu (China) Co., Ltd., Xi’an, China) with splitless injection, MRM acquisition mode. The capillary column Rxi-5Sil Ms (30 m × 0.25 mm × 0.25 μm) was used for separations. Helium (99.999%) was used as the carrier gas. The oven temperature program was as follows: initial temperature of 50 °C was held for 2 min, then increased to 250 °C at a rate of 20 °C/min and held for 3 min, and finally increased to 300 °C at a rate of 5 °C/min and held for 5 min.

### 2.6. Quality Control

All analytical procedures were monitored with strict quality assurance and quality control measures. Quantitation was performed using an external standard calibration method (seven-point calibration: 2, 10, 50, 100, 200, 500 and 1000 μg/L), and correlation coefficients (R^2^) for the calibration curves that were all greater than 0.996. The limit of detection (LOD) was calculated as three times of standard deviation of the blank. The LODs of NAP, ACY, ACE, FLU, PHE, ANT, FLA and PYR were 0.02, 0.80, 0.60, 0.12, 0.04, 0.16, 0.12 and 0.16 μg/kg dw, and those of BaA, CHR, BbF, BkF, BaP, InP, DBA and BgP were 0.18, 0.08, 0.16, 0.20, 0.20, 0.06, 0.06 and 0.10 μg/kg dw, respectively. The recoveries of NAP, ACY, ACE, FLU, PHE, ANT, FLA and PYR were 118 ± 1.7%, 117 ± 0.5%, 119 ± 3.5%, 112 ± 0.8%, 109 ± 6.7%, 94 ± 6.4%, 109 ± 1.1% and 110 ± 5.9%, and those of BaA, CHR, BbF, BkF, BaP, InP, DBA and BgP were 95 ± 2.8%, 96 ± 3.5%, 98 ± 9.1%, 93 ± 7.6%, 65 ± 8.9%, 93 ± 6.3%, 94 ± 3.9% and 80 ± 9.5%, respectively.

### 2.7. Ecological Risk of PAHs in Soils

PAHs accumulated in soils may enter water bodies and plants, posing a potential ecological risk. A risk quotient (RQ) was used to assess ecological risk of some organic substances. The negligible concentrations (NCs) and the maximum permissible concentrations (MPCs) of PAHs in soils were used as the quality values in the medium [16]. Therefore, RQ_(NCs)_ and RQ_(MPCs)_ were defined as follows:

$${\mathrm{RQ}}_{\mathrm{NCs}}=\frac{C_{\mathrm{PAHs}}}{C_{\mathrm{QV}(\mathrm{NCs})}}$$

$${\mathrm{RQ}}_{\mathrm{MPCs}}=\frac{C_{\mathrm{PAHs}}}{C_{\mathrm{QV}(\mathrm{MPCs})}}$$

where C_QV(NCs)_ was the quality values of the NCs of PAHs in the medium and C_QV(MPCs)_ was the quality values of the MPCs of PAHs in the medium. The RQ_∑PAHs_, RQ_∑PAHs(NCs)_ and RQ_∑PAHs(MPCs)_ were defined as follows:

$${\mathrm{RQ}}_{\sum \mathrm{PAHs}}=\sum_{i=1}^{16}{\mathrm{RQ}}_{i},\;{\mathrm{RQ}}_{i}\;\geq \;1$$

$${\mathrm{RQ}}_{\sum \mathrm{PAHs}(\mathrm{NCs})}=\sum_{i=1}^{16}{\mathrm{RQ}}_{i(\mathrm{NCs})},\;{\mathrm{RQ}}_{i(\mathrm{NCs})}\;\geq \;1$$

$${\mathrm{RQ}}_{\sum \mathrm{PAHs}(\mathrm{MPCs})}=\sum_{i=1}^{16}{\mathrm{RQ}}_{i(\mathrm{MPCs})},\;{\mathrm{RQ}}_{i(\mathrm{MPCs})}\;\geq \;1$$

Based on the ecosystem risk assessment of 16 individual PAHs, RQ_(NCs)_ and RQ_(MPCs)_ of individual PAHs which were not less than 1 were added to calculate the RQ_∑PAHs(NCs)_ and RQ_∑PAHs(MPCs)_ of ∑PAHs. RQ_(NCs)_ < 1.0 indicated that the single PAHs might be of negligible concern, RQ_(MPCs)_ > 1.0 would indicate that the contamination of the single PAHs posed high risk, and RQ_(NCs)_ > 1.0 and RQ_(MPCs)_ < 1.0 indicated that the contamination of the single PAHs was of moderate risk.

### 2.8. Toxicity Assessment of PAHs in Soils

PAHs can be absorbed by humans through the skin and respiratory tract, and they may cause skin cancer, lung cancer and other diseases. Exposure to PAHs in the environment for a long time may cause chronic poisoning. Toxic equivalency factors (TEFs) were used to estimate the exposure risks posed by individual and total PAHs to human health. The toxicities of PAHs in sampling sites were evaluated BaP equivalent concentration (BaPeq). The TEFs for the 16 PAHs were calculated according to USEPA and Nisbet and LaGoy [10,17]. The total toxicity equivalency concentrations (BaPeq) were calculated using the following equation:

$$\sum \mathrm{BaPeq}=\sum C_{i}\times {\mathrm{TEF}}_{i}$$

where C_i_ is the concentration of individual PAHs and TEF_i_ is the corresponding toxic equivalency factor.

### 2.9. Properties Analysis

Soil pH was measured (soil: water 1:2.5 *w*/*v*) by using a pH-meter (pHS-3B, Leici, Shanghai, China) and the soil organic carbon contents were determined by the Walkey-Black method [18].

## 3. Results and Discussion

### 3.1. Characteristics of PAHs Concentrations in Soils

As shown in Table 2, all 16 priority PAHs were detected in petroleum-contaminated soils, indicating that PAHs were ubiquitous pollutants in the tested soil environment. The total concentrations of ∑16PAHs in top layer soils (0–10 cm), middle layer soils (10–30 cm), and bottom layer soils (30–50 cm) ranged from 1010.67 to 18,068.80, 495.85 to 9868.56 and 213.16 to 12,552.53 μg/kg, with an average of 5502.44, 2296.94 and 2203.88 μg/kg, respectively. Moreover, the human carcinogen compounds (BaA, CHR, BbF, BkF, BaP, DBA and InP) were also investigated in petroleum-contaminated soils, and the results are presented in Table 2. The highest total carcinogenic PAHs (∑7PAHs) were distributed in top layer soils with a range of 223.97–4642.40 μg/kg (mean: 1832.55 μg/kg), followed by the bottom layer soils (range: 23.89–6588.26 μg/kg, mean: 1039.09 μg/kg) and the middle layer soils (range: 85.71–3466.19 μg/kg, mean: 921.45 μg/kg). Among these human carcinogen compounds, BaP is a typical PAH which is of greatest interest in terms of potential cancer hazard [19]. BaP concentration varied in a range of 10.40–225.40 μg/kg, N.D.–191.80 μg/kg, N.D.–911.71 μg/kg for the top layer soils, middle layer soils, and bottom layer soils, respectively (Table 2).

According to the European classification system of soil contamination [20], the PAHs pollution in soils was divided into four grades. A ∑16PAHs soil concentration below 200 μg/kg indicates no polluted, a concentration of 200–600 μg/kg represents lightly polluted, and a soil concentration of 600–1000 μg/kg represents moderately polluted. Concentrations over 1000 μg/kg would be indicative of heavy pollution. According to this classification standard, it is worthwhile to note that ∑16PAHs in petroleum-contaminated soils were 2.2–5.5 times higher than the standard level (1000 μg/kg) of heavy polluted. It indicated that the petroleum-contaminated soils stored great amount of PAHs and regulatory measures are needed to prevent the areas from turning into pollution sources, which would transfer PAHs into the air or groundwater in the region.

In addition, a comparison of ∑PAHs concentrations in soils from different cities worldwide is given in Table 3, where it can be seen that the mean concentration of ∑16PAHs in petroleum-contaminated soils was much lower than that in urban soil from London (UK) and garden soil from New York (USA). However, it was higher than that in different types of soil from some Chinese region such as Xianyang, Shanghai, Nanjing, Tianjin, Jilin, Momoge Wetland and Yangtze River Delta, as well as from Dhanbad (India), New Orleans (USA), Ulsan (Korea), Viseu (Portugal) and Isfahan (Iran). The results indicate people should be cautious about the environmental quality of the petroleum-contaminated soils.

### 3.2. Characteristics of the PAHs Distribution in Soils

**Figure 2:**
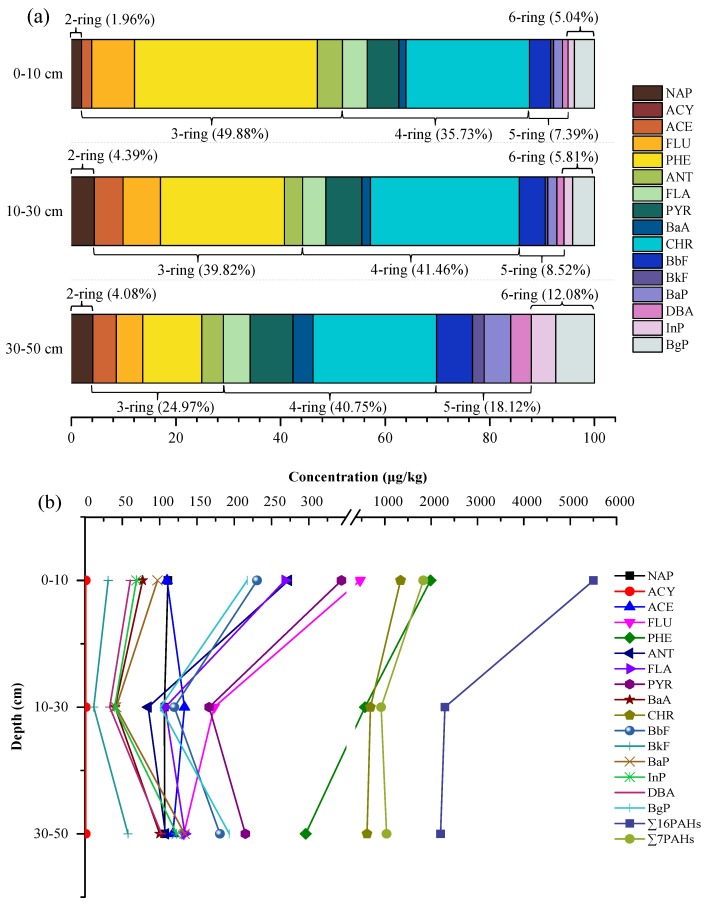
Distribution of (**a**) PAHs composition in different layers of soil and (**b**) PAHs component in different layers of soil.

PAHs represent complex chemicals which consist of multiple aromatic rings. Based on the number of aromatic rings, the 16 PAHs are divided into five groups: 2-ring, 3-ring, 4-ring, 5-ring, 6-ring PAHs. The distribution pattern of the 16 PAHs is shown in Figure 2a. It can be seen that the sequence of the PAHs proportion in top layer soils (0–10 cm) was detected as 3-ring (49.88%) > 4-ring (35.73%) > 5-ring (7.39%) > 6-ring (5.04%) > 2-ring (1.96%). Correspondingly, the sequence of the PAHs proportion in middle layer soils (10–30 cm) was detected as 4-ring (41.46%) > 3-ring (39.82%) > 5-ring (8.52%) > 6-ring (5.81%) > 2-ring (4.39%). In addition, the distribution pattern of PAHs in bottom layer soils and middle layer soils is same. It is obvious that the soil samples in different layers had the same PAHs compositions in terms of the number of aromatic rings. The 3-ring and 4-ring PAHs were analyzed as the most prominent components in all soil samples. Moreover, due to their high volatility, 2-ring PAHs was lower in the top and bottom layer soils, but higher in middle layer soils. The 5-ring and 6-ring PAHs levels increased with the increasing depth, the reason being that they have high hydrophobicity and molecular mass, so they can accumulate more easily by adsorption on soil organic matter.

The vertical distribution of PAHs in petroleum-contaminated soils was assessed from the soil samples collected from vertical sections at three depths in the sampling areas. The results of the vertical distribution profile of PAHs component are shown in Figure 2b. It is expected that ∑16PAHs would gradually decrease with the increasing depth, from the top layer (0–10 cm) to the bottom layer (30–50 cm), resulting in decreasing ∑16PAHs from 5502.44 μg/kg to 2203.88 μg/kg. Compared to the top layer soils, the ∑16PAHs in 30–50 cm depth decreased by 59.95% in the sample area. The vertical distribution profile of ∑7PAHs is similar to that of ∑16PAHs. It is also found that ∑7PAHs would gradually decrease with the increasing depth, and the concentration decreased from 1832.55 μg/kg (0–10 cm) to 1039.09 μg/kg (30–50 cm).

What’s more, the results of the individual PAHs concentration in different vertical sections are also shown in Figure 2b. The vertical distribution characteristics of individual PAHs appeared to be different. The highest concentration of Nap, FLU, PHE, ANT, FLA, PYR, CHR, BbF and BgP were obtained in the top layer soils (0–10 cm). More accurately, FLU, PHE and CHR were found to be the most prominent compounds in all soil samples.

### 3.3. Correlation Analysis

The relationships between ∑16PAHs, soil organic matter (SOM) and pH were investigated in the present study (Table 4). Soil pH can affect the residual of PAHs in soils [33]. However, no significant correlation relationships between soil pH and PAHs were found in the present study, implying soil pH was not a key factor in the soil PAHs levels. SOM is considered to be key factor influencing the concentration of PAHs in soils. Nam et al. [34] reported that, in an environment where there is continuous input of fresh PAHs, a lack of correlation should be expected, at least until equilibrium is reached. In this study, good correlation existed between SOM and the concentration of 16 PAHs was found, suggesting that soil PAHs were close to steady state and in equilibrium with SOM.

### 3.4. Source Identification of PAHs in Soils

Understanding the sources of PAHs is crucial to determine how PAHs are carried into the environment. Generally, the diagnostic ratios method was the most widely used to distinguish between the sources of PAHs in the soil ecosystem. Ratios such as low molecular weight (2–3 rings, LMW)/high molecular weight (≥4 rings, HMW), FLA/(FLA + PYR), BaA/(BaA + CHR) and ANT/(ANT + PHE) have been reported in many studies. For example, the ratio of LMW/HMW < 1 indicates pyrogenic source, while the ratio >1 indicates petrogenic source [35]. A ratio of FLA/(FLA + PYR) < 0.4 indicates a petroleum source, a ratio between 0.4–0.5 indicates a fossil fuel combustion source, and a ratio >0.5 indicates coal/wood/grass combustion source [36]. For BaA/(BaA + CHR), the ratio < 0.2 indicates a petroleum source, the ratio between 0.2–0.35 indicates a mixed source, and the ratio >0.35 indicates a combustion source [37]. Values of ANT/(ANT + PHE) ratio are <0.1 and >0.1 indicative of petroleum and combustion sources, respectively [38].

**Figure 3:**
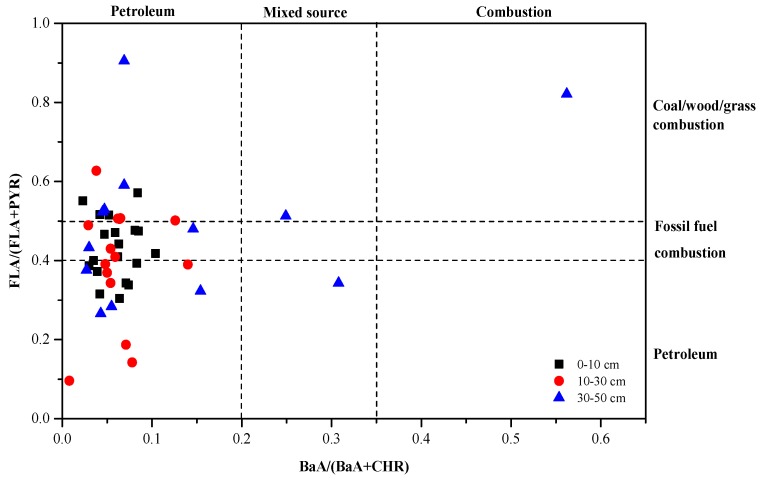
The ratios for BaA/(BaA + CHR) versus FLA/(FLA + PYR) in soils.

In this study, the diagnostic ratios of FLA/(FLA + PYR) and BaA/(BaA + CHR) were used to distinguish the possible PAHs origins in petroleum-contaminated soils. The ratios for BaA/(BaA + CHR) versus FLA/(FLA + PYR) are shown in Figure 3, where the BaA/(BaA + CHR) values for 95% of the samples are <0.2, while the FLA/(FLA + PYR) values for 75% of the samples are <0.5. This suggests that the PAHs in soil samples come from petroleum sources and only a small quantity of them comes from combustion sources.

### 3.5. Ecological Risk of PAHs in Soils

The assessment results of ecological risk of PAHs in petroleum-contaminated soils based on risk quotient are given in Table 5. The mean values of RQ_(NCs)_ for most individual PAHs were found to be greater than 1, with the exception of ACY (0.00), BkF (0.49) and InP (0.67). The mean values of calculated RQ_(MPCs)_ for FLU, PHE and PYR were greater than 1, implying that these three PAHs had high ecological risk to aquatic/soil organisms. The mean value of calculated RQ_∑PAHs(NCs)_ was above 800, while the mean value of calculated RQ_∑PAHs(MPCs)_ was higher than 1, suggesting that the total of PAHs in petroleum-contaminated soils was assigned a high ecological risk level. It is worth noting that though low molecular PAHs are less mutagenic and carcinogenic than high molecular PAHs, it can be seen from Table 5 that ecosystem risk associated with low and molecular PAHs is actually very high. Therefore, control and preventive measures should be implemented to decrease the contamination associated with 2-ring, 3-ring and 4-ring PAHs.

### 3.6. Toxicity Potential of PAHs in Soils

Toxic equivalency quantities (TEQs) calculated as toxic equivalency factors (TEFs) are given in Table 6. As shown in Table 6, the TEQs of ∑16PAHs in top layer soils (0–10 cm), middle layer soils (10–30 cm), and bottom layer soils (30–50 cm) ranged from 16.59 to 303.50, 2.59 to 165.19 and 0.21 to 1452.16 μg/kg, with an average of 220.31, 106.25 and 292.48 μg/kg, respectively. Meanwhile, the TEQs of ∑7PAHs in soils of 0–10 cm, 10–30 cm and 30–50 cm ranged from 11.90 to 277.19, 3.23 to 277.28 and 0.24 to 1436.87 μg/kg, with an average of 212.13, 103.14 and 288.46 μg/kg, respectively. It is found that the TEQs of ∑7PAHs were very close to that of ∑16PAHs, indicating that the ∑7PAHs were the major carcinogenic contributor to the TEQs of ∑16PAHs. According to the Canadian soil quality guidelines for the protection of environmental and human health, the safe value of the TEQs of ∑7PAHs in soils is 600 μg/kg [39]. All the soil samples in this study were below the safe value. In addition, the TEQs of ∑7PAHs were much higher than that in soils of Hunpu (52.31 μg/kg) [35], Xinzhou (34 μg/kg) [40], Liaohe estuary (30.0 μg/kg) [41] and Yellow River Delta (11.92 μg/kg) [42]; while lower than that in soils of Xi’an (421.05 μg/kg) [43]. These indicated that PAHs in petroleum-contaminated soils presented relatively high toxicity.

## 4. Conclusions

The present study indicates the concentration and potential ecological risk of PAHs in different layers of soil in the petroleum-contaminated areas of Loess Plateau in China. The following conclusions can be drawn from the results of this study:

1. The concentrations of ∑16PAHs and ∑7PAHs ranged from 1010.67 to 18,068.80 μg/kg and from 223.97 to 4642.40 μg/kg in top layer soils (0–10 cm), from 495.85 to 9868.56 μg/kg and from 85.71 to 3466.19 μg/kg in middle layer soils (10–30 cm), and from 213.16 to 12,552.53 μg/kg and from 23.89 to 6588.26 μg/kg in bottom layer soils (30–50 cm), respectively. In general, the Σ16PAHs presented heavy pollution.
2. It is noted that ∑16PAHs gradually decreases over increasing depth. The most prominent compositions analyzed in all soil samples were the 3-ring and 4-ring PAHs, and FLU, PHE and CHR were dominant compounds.
3. There was no significant correlation between ∑16PAHs and soil pH, but noteworthy correlations between ∑16PAHs and SOM.
4. The source of PAHs in the studied soils is primarily petroleum, and only a small quantity of these originates from combustion.

The soil ecological risk and toxicity assessment indicate that petroleum-contaminated soils present a relatively high ecological risk level and toxicity. Therefore, it is urgent for the government to take corresponding measures to reverse the ecological state of petroleum-contaminated soils. We will continue to study the ecotoxicity of PAHs, including their effect on soil microbial communities. At the same time, we will select different agents for the remediation of petroleum-contaminated soils.

## Figures and Tables

**Table 1:** Basic information of the sampling sites in this study.

Sites	City	Detailed Information of the Sampling Sites	Altitude (m)	Geographic Coordinates		Era of Close-By Oil Wells
				Latitude (N)	Longitude (E)	
S1	Yan’an	Hua Zi Ping Town, Ansai District	1109	36°59′00″	109°14′22″	1983s
S2		Yu Jia Ping Town, Zichang County	1322	37°04′36″	109°24′25″	2002s
S3		Yu Jia Ping Town, Zichang County	1365	37°04′45″	109°24′41″	2003s
S4		Hei Jia Bao Town, Yanchang County	860	36°39′09″	109°53′05″	1980s
S5		Wu Cang Bao Town, Wuqi County	1522	36°58′33″	108°12′58″	2013s
S6	Yulin	Xiao He Town, Jingbian County	1475	37°25′02″	108°57′04″	2009s
S7		Xiao He Town, Jingbian County	1415	37°22′40″	109°00′45″	1989s
S8		Zhong Shan Jian Town, Jingbian County	1518	37°19′52″	108°27′56″	2015s
S9		Zhong Shan Jian Town, Jingbian County	1630	37°20′46″	108°26′32″	2015s
S10		Hao Tan Village, Dingbian County	1374	37°29′58″	108°11′36″	2012s
S11		An Bian Town, Dingbian County	1456	37°29′58″	108°00′25″	2012s
S12		Zhuan Jing Town, Dingbian County	1430	37°30′39″	107°54′26″	1992s
S13		Zhuan Jing Town, Dingbian County	1452	37°30′41″	107°54′27″	2016s
S14		Zhuan Jing Town, Dingbian County	1564	37°28′08″	107°42′12″	2003s
S15	Qingyang	Qiao He Village, Huachi County	1644	36°35′44″	108°04′39″	1980s
S16		Qiao He Village, Huachi County	1502	36°33′08″	108°04′52″	2008s
S17		Yue Le Town, Huachi County	1316	36°21′27″	108°00′59″	2012s
S18		Bai Ma Pu Town, Qingcheng County	1422	35°53′40″	107°43′51″	2004s
S19		Mi Qiao Village, Ning County	1380	35°28′44″	108°14′55″	2010s
S20		Mi Qiao Village, Ning County	1396	35°29′18″	108°16′44″	2012s

**Table 2:** Concentrations of individual PAHs, ∑16PAHs and ∑7PAHs in the samples from different layers of soil.

PAH Compounds	Abbreviation	Ring of Numbers	0–10 cm (μg/kg)			10–30 cm (μg/kg)			30–50 cm (μg/kg)		
			Range	Mean	Median	Range	Mean	Median	Range	Mean	Median
Naphthalene	NAP	2-ring	64.46–196.55	111.17	94.52	59.61–196.45	106.40	83.88	59.14–167.77	106.88	96.30
Acenaphthylene	ACY	3-ring	N.D.	N.D.	N.D.	N.D.	N.D.	N.D.	N.D.–31.05	N.D.	N.D.
Acenaphthene	ACE	3-ring	N.D.–157.98	109.97	119.69	N.D.–466.99	133.41	113.21	N.D.–390.27	117.72	86.98
Fluorene	FLU	3-ring	44.48-2383.70	465.33	169.63	57.77–1326.62	173.08	88.57	32.71–598.08	132.46	88.57
Phenanthrene	PHE	4-ring	127.81–8052.24	1984.77	1009.95	108.04–5857.47	574.11	203.14	68.82–895.03	295.71	187.66
Anthracene	ANT	3-ring	N.D.–1317.29	272.76	104.87	N.D.–743.06	84.64	38.75	N.D.–593.61	107.96	40.31
Fluoranthene	FLA	4-ring	23.09–867.22	267.97	195.62	N.D.–538.54	107.55	55.73	9.76–951.35	133.81	27.75
Pyrene	PYR	4-ring	34.62–872.94	343.67	300.32	20.95–596.16	166.36	118.32	N.D.–2404.15	214.93	59.72
Benzo(a)anthracene	BaA	4-ring	N.D.–185.51	77.19	72.97	N.D.–153.01	40.27	24.67	N.D.–507.76	100.25	31.06
Chrysene	CHR	4-ring	116.65–3796.53	1340.26	1142.36	42.23–2688.38	690.65	367.48	N.D.–2976.13	618.21	391.56
Benzo(b)fluoranthene	BbF	5–ring	27.08–572.11	230.53	167.74	12.00–381.80	119.70	88.44	N.D.–1243.23	180.78	95.57
Benzo(k)fluoranthene	BkF	5–ring	N.D.–67.05	31.13	21.57	N.D.–21.69	11.71	10.72	N.D.–231.93	57.58	15.92
Benzo(a)pyrene	BaP	5–ring	10.40–225.40	97.23	73.11	N.D.–191.80	41.86	25.61	N.D.–911.71	133.54	48.54
Indeno(1,2,3–c,d)pyrene	InP	6–ring	N.D.–151.52	68.92	58.50	N.D.–82.37	39.72	37.09	N.D.–467.10	122.89	30.75
Dibenzo(a,h)anthracene	DBA	5–ring	N.D.–102.03	60.72	60.21	N.D.–80.00	33.23	31.98	N.D.–250.40	102.59	90.71
Benzo(g,h,i)perylene	BgP	6–ring	N.D.–433.26	217.30	217.56	N.D.–272.75	101.12	98.00	N.D.–752.43	193.43	118.27
∑16PAHs ∑7PAHs			1010.67–18068.80	5502.44	4030.25	495.85–9868.56	2296.94	1411.88	213.16–12552.53	2203.88	1070.47
			223.97–4642.40	1832.55	1791.17	85.71–3466.19	921.45	576.88	23.89–6588.26	1039.09	553.09

N.D.: Not Detected; ∑16PAHs: the total of sixteen PAHs; ∑7PAHs: the sum of seven carcinogenic PAHs including BaA, CHR, BbF, BkF, BaP, DBA and InP.

**Table 3:** Concentration comparison of ΣPAHs in soils from worldwide different cities.

District	Soil Types	Depth (cm)	Number of PAHs	Mean (μg/kg)	References
Loess Plateau, China	petroleum-contaminated soil	0–10	16	5502.44	This study
Xianyang, China	vegetable soil	0–25	16	210.31	Wang et al. [7]
Shanghai, China	urban soil	0–20	16	1970	Wang et al. [21]
Nanjing, China	urban soil	0–5	16	3330	Wang et al. [22]
Tianjin, China	farmland soil	0–20	16	941.27	Shi et al. [23]
Jilin, China	agricultural soil	0–20	16	877.23	Chen et al. [24]
Momoge Wetland, China	wetland soil	0–10	16	96	Xu et al. [25]
Yangtze River Delta region, China	industrial areas soil	0–20	16	471.3	Wang et al. [26]
Dhanbad, India	urban traffic soil	0–5	13	3488	Suman et al. [15]
London, UK	urban soil	5–20	16	18,000	Vane et al. [27]
New Orleans, USA	urban soil	0–2.5	16	2927	Mielke et al. [28]
New York, USA	garden soil	0–10	16	14,200	Marquez-Bravo et al. [29]
Ulsan, Korea	industrial, urban, and rural soil	0–5	16	960	Kwon et al. [30]
Viseu, Portugal	urban soil	0–10	16	169	Cachada et al. [31]
Isfahan, Iran	urban soil	1–5	16	2000.56	Moore et al. [32]

**Table 4:** Correlation analysis between ∑16PAHs, soil organic matter and pH.

	SOM (0–10 cm)	SOM (10–30 cm)	SOM (30–50 cm)	pH (0–10 cm)	pH (10–30 cm)	pH (30–50 cm)	∑16PAHs (0–10 cm)	∑16PAHs (10–30 cm)	∑16PAHs (30–50 cm)
SOM (0–10 cm)	1								
SOM (10–30 cm)	0.439	1							
SOM (30–50 cm)	0.138	0.821 **	1						
pH (0–10 cm)	0.128	−0.227	−0.285	1					
pH (10–30 cm)	0.065	−0.162	−0.083	0.720 **	1				
pH (30–50 cm)	−0.005	−0.335	−0.338	0.516 *	0.676 **	1			
∑16PAHs (0–10 cm)	0.810 **	0.510 *	0.234	0.168	0.171	0.021	1		
∑16PAHs (10–30 cm)	0.383	0.812 **	0.567 **	−0.252	0.016	−0.213	0.506 *	1	
∑16PAHs (30–50 cm)	0.075	0.439	0.780 **	−0.075	0.152	−0.007	0.170	0.305	1

* Correlation is significant at *p* < 0.05 (two-tailed); ** Correlation is significant at *p* < 0.01 (two-tailed).

**Table 5:** Descriptive statistics of RQ(NCs) and RQ(MPCs) of PAHs in soils (µg/kg).

PAHs	NCs	MPCs	RQ_(NCs)_			RQ_(MPCs)_		
			0–10 cm	10–30 cm	30–50 cm	0–10 cm	10–30 cm	30–50 cm
NAP	1.4	140	79.41	76.00	76.35	0.79	0.76	0.76
ACY	1.2	120	0.00	0.00	0.00	0.00	0.00	0.00
ACE	1.2	120	91.64	111.18	98.10	0.92	1.11	0.98
FLU	1.2	120	387.77	144.23	110.38	3.88	1.44	1.10
PHE	5.1	510	389.17	112.57	57.98	3.89	1.13	0.58
ANT	1.2	120	227.30	70.53	89.96	2.27	0.71	0.90
FLA	26	2600	10.31	4.14	5.15	0.10	0.04	0.05
PYR	1.2	120	286.39	138.63	179.11	2.86	1.39	1.79
BaA	2.5	250	30.87	16.11	40.10	0.31	0.16	0.40
CHR	107	10700	12.53	6.45	5.78	0.13	0.06	0.06
BbF	2.5	250	92.21	47.88	72.31	0.92	0.48	0.72
BkF	24	2400	1.30	0.49	2.40	0.01	0.00	0.02
BaP	2.6	260	37.39	16.10	51.36	0.37	0.16	0.51
InP	59	5900	1.17	0.67	2.08	0.01	0.01	0.02
DBA	2.6	260	23.35	12.78	39.46	0.23	0.13	0.39
BgP	75	7500	2.90	1.35	2.58	0.03	0.01	0.03
∑16PAHs			1673.72	757.96	833.10	12.91	5.07	2.89

**Table 6:** Toxic equivalence quantities (TEQs) of PAHs in petroleum-contaminated soils (μg/kg).

PAHs	TEFs	0–10 cm (μg/kg)			10–30 cm (μg/kg)			30–50 cm (μg/kg)		
		Min	Max	Mean	Min	Max	Mean	Min	Max	Mean
NAP	0.001	0.06	0.20	0.11	0.06	0.20	0.11	0.06	0.17	0.11
ACY	0.001	N.D.	N.D.	N.D.	N.D.	N.D.	N.D.	N.D.	0.03	N.D.
ACE	0.001	N.D.	0.16	0.11	N.D.	0.47	0.13	N.D.	0.39	0.12
FLU	0.001	0.04	2.38	0.47	0.06	1.33	0.17	0.03	0.60	0.13
PHE	0.001	0.13	8.05	1.98	0.11	5.86	0.57	0.07	0.90	0.30
ANT	0.01	N.D.	13.17	2.73	N.D.	7.43	0.85	N.D.	5.94	1.08
FLA	0.001	0.02	0.87	0.27	N.D.	0.54	0.11	0.01	0.95	0.13
PYR	0.001	0.03	0.87	0.34	0.02	0.60	0.17	N.D.	2.40	0.21
BaA	0.1	N.D.	18.55	7.72	N.D.	15.30	4.03	N.D.	50.78	10.02
CHR	0.01	1.17	37.97	13.40	0.42	26.88	6.91	N.D.	29.76	6.18
BbF	0.1	2.71	57.21	23.05	1.20	38.18	11.97	N.D.	124.32	18.08
BkF	0.1	N.D.	6.70	3.11	N.D.	2.17	1.17	N.D.	23.19	5.76
BaP	1	10.40	225.40	97.23	N.D.	191.80	41.86	N.D.	911.71	133.54
InP	0.1	N.D.	15.15	6.89	N.D.	8.24	3.97	N.D.	46.71	12.29
DBA	1	N.D.	102.03	60.72	N.D.	80.00	33.23	N.D.	250.40	102.59
BgP	0.01	N.D.	4.33	2.17	N.D.	2.73	1.01	N.D.	7.52	1.93
∑16PAHs		16.59	303.50	220.31	2.59	165.19	106.25	0.21	1452.16	292.48
∑7PAHs		11.90	277.19	212.13	3.23	277.28	103.14	0.24	1436.87	288.46

## References

[1] Gu Y.G., Ke C.L., Liu Q., Lin Q. (2016). Polycyclic aromatic hydrocarbons (PAHs) in sediments of Zhelin Bay, the largest mariculture base on the eastern Guangdong coast, South China: Characterization and risk implications. Mar. Pollut. Bull..

[2] Johnsen A.R., Wick L.Y., Harms H. (2005). Principles of microbial PAH-degradation in soil. Environ. Pollut..

[3] Jiang Y., Yves U.J., Sun H., Hu X., Zhan H., Wu Y. (2016). Distribution, compositional pattern and sources of polycyclic aromatic hydrocarbons in urban soils of an industrial city, Lanzhou, China. Ecotoxicol. Environ. Saf..

[4] Yin C.Q., Jiang X., Yang X.L., Bian Y.R., Wang F. (2008). Polycyclic aromatic hydrocarbons in soils in the vicinity of Nanjing, China. Chemosphere.

[5] Kamal A., Cincinelli A., Martellini T., Malik R.N. (2015). A review of PAH exposure from the combustion of biomass fuel and their less surveyed effect on the blood parameters. Environ. Sci. Pollut. Res..

[6] Wild S.R., Jones K.C. (1995). Polynuclear aromatic hydrocarbons in the United Kingdom environment: A preliminary source inventory and budget. Environ. Pollut..

[7] Wang L.J., Xu X., Lu X.W. (2016). Composition, source and potential risk of polycyclic aromatic hydrocarbons (PAHs) in vegetable soil from the suburbs of Xianyang City, Northwest China: A case study. Environ. Earth Sci..

[8] Thorsen W.A., Cope W.G., Shea D. (2004). Bioavailability of PAHs: Effects of soot carbon and PAH source. Environ. Sci. Technol..

[9] Cornelissen G., Breedveld G.D., Kalaitzidis S., Christanis K., Kibsgaard A., Oen A.M.P. (2006). Strong sorption of native PAHs to pyrogenic and unburned carbonaceous geosorbents in sediments. Environ. Sci. Technol..

[10] United States Environmental Protection Agency (USEPA) (1998). Guidelines for ecological risk assessment. Fed. Regist..

[11] Shamilishvily G., Abakumov E., Gabov D. (2018). Polycyclic aromatic hydrocarbon in urban soils of an Eastern European megalopolis: Distribution, source identification and cancer risk evaluation. Solid Earth.

[12] Tong R.P., Yang X.Y., Su H.R., Pan Y., Zhang Q.Z., Wang J., Long M.C. (2018). Levels, sources and probabilistic health risks of polycyclic aromatic hydrocarbons in the agricultural soils from sites neighboring suburban industries in Shanghai. Sci. Total Environ..

[13] Liu H., Yu X.L., Liu Z.R., Sun Y. (2018). Occurrence, characteristics and sources of polycyclic aromatic hydrocarbons in arable soils of Beijing, China. Ecotoxicol. Environ. Saf..

[14] Wang Y.P., Liang J.D., Wang J.X., Gao S. (2018). Combining stable carbon isotope analysis and petroleum-fingerprinting to evaluate petroleum contamination in the Yanchang oilfield located on loess plateau in China. Environ. Sci. Pollut. Res..

[15] Suman S., Sinha A., Tarafdar A. (2016). Polycyclic aromatic hydrocarbons (PAHs) concentration levels, pattern, source identification and soil toxicity assessment in urban traffic soil of Dhanbad, India. Sci. Total Environ..

[16] Kalf D.F., Crommentuijn T., Van De Plassche E.J. (1997). Environmental quality objectives for 10 polycyclic aromatic hydrocarbons (PAHs). Ecotoxicol. Environ. Saf..

[17] Nisbet I.C.T., LaGoy P.K. (1992). Toxic equivalency factors (TEFs) for polycyclic aromatic hydrocarbons (PAHs). Regul. Toxicol. Pharmacol..

[18] Sun C., Liu J., Wang Y., Sun L., Yu H. (2013). Multivariate and geostatistical analyses of the spatial distribution and sources of heavy metals in agricultural soil in Dehui, Northeast China. Chemosphere.

[19] Jiao H.H., Wang Q., Zhao N.N., Jin B., Zhuang X.L., Bai Z.H. (2017). Distributions and sources of Polycyclic Aromatic Hydrocarbons (PAHs) in soils around a chemical plant in Shanxi, China. Int. J. Environ. Res. Public Health.

[20] Maliszewska-Kordybach B. (1996). Polycyclic aromatic hydrocarbons in agricultural soils in Poland: Preliminary proposals for criteria to evaluate the level of soil contamination. Appl. Geochem..

[21] Wang X.T., Miao Y., Zhang Y., Li Y.C., Wu M.H., Yu G. (2013). Polycyclic aromatic hydrocarbons (PAHs) in urban soils of the megacity Shanghai: Occurrence, source apportionment and potential human health risk. Sci. Total Environ..

[22] Wang C.H., Wu S.H., Zhou S.L., Wang H., Li B.J., Chen H., Yu Y.N., Shi Y.X. (2015). Polycyclic aromatic hydrocarbons in soils from urban to rural areas in Nanjing: Concentration, source, spatial distribution, and potential human health risk. Sci. Total Environ..

[23] Shi R.G., Xu M.M., Liu A.F., Tian Y., Zhao Z.S. (2017). Characteristics of PAHs in farmland soil and rainfall runoff in Tianjin, China. Environ. Monit. Assess..

[24] Chen Y.A., Zhang J.Q., Zhang F., Li F.X., Zhou M. (2018). Polycyclic aromatic hydrocarbons in farmland soils around main reservoirs of Jilin Province, China: Occurrence, sources and potential human health risk. Environ. Geochem. Health.

[25] Xu J.L., Wang H.X., Sheng L.X., Liu X.J., Zheng X.X. (2017). Distribution characteristics and risk assessment of Polycyclic Aromatic Hydrocarbons in the Momoge Wetland, China. Int. J. Environ. Res. Public Health.

[26] Wang J., Zhang X.F., Ling W.T., Liu R., Liu J., Kang F.X., Gao Y.Z. (2017). Contamination and health risk assessment of PAHs in soils and crops in industrial areas of the Yangtze River Delta region, China. Chemosphere.

[27] Vane C.H., Kim A.W., Beriro D., Cave M.R., Knights K., Moss-Hayes V., Nathanail P.C. (2014). Polycyclic aromatic hydrocarbons (PAH) and polychlorinated biphenyls (PCB) in urban soils of Greater London, UK. Appl. Geochem..

[28] Mielke H.W., Wang G.D., Gonzales C.R., Powell E.T., Le B., Quach V.N. (2004). PAHs and metals in the soils of inner-city and suburban New Orleans, Louisiana, USA. Environ. Toxicol. Pharmacol..

[29] Marquez-Bravo L.G., Briggs D., Shayler H., McBride M., Lopp D., Stone E., Ferenz G., Bogdan K.G., Mitchell R.G., Spliethoff H.M. (2016). Concentrations of polycyclic aromatic hydrocarbons in New York City community garden soils: Potential sources and influential factors. Environ. Toxicol. Chem..

[30] Kwon H.O., Choi S.D. (2014). Polycyclic aromatic hydrocarbons (PAHs) in soils from a multi-industrial city, South Korea. Sci. Total Environ..

[31] Cachada A., Pato P., Tocha-Santos T., Ferreira da Silva E., Duarte A.C. (2012). Levels, sources and potential human health risks of organic pollutants in urban soils. Sci. Total Environ..

[32] Moore F., Akhbarizadeh R., Keshavarzi B., Khabazi S., Lahijanzadeh A., Kermani M. (2015). Ecotoxicological risk of polycyclic aromatic hydrocarbons (PAHs) in urban soil of Isfahan metropolis, Iran. Environ. Monit. Assess..

[33] Jiao W.T., Wang T.Y., Lu Y.L., Chang A., Chen W.P. (2013). Multi-factors influencing the spatial distribution of polycyclic aromatic hydrocarbons in soils surrounding drinking water protection zone. J. Environ. Sci..

[34] Nam J.J., Thomas G.O., Jaward F.M., Steinnes E., Gustafsson O., Jones K.C. (2008). PAHs in background soils from Western Europe: Influence of atmospheric deposition and soil organic matter. Chemosphere.

[35] Zhang J., Yang J.C., Wang R.Q., Hou H., Du X.M., Fan S.K., Liu J.S., Dai J.L. (2013). Effects of pollution sources and soil properties on distribution of polycyclic aromatic hydrocarbons and risk assessment. Sci. Total Environ..

[36] Yunker M.B., Macdonald R.W., Vingarzan R., Mitchell R.H., Goyette D., Sylvestre S. (2002). PAHs in the Fraser River basin: A critical appraisal of PAH ratios as indicators of PAH source and composition. Org. Geochem..

[37] Christensen E.R., Bzdusek P.A. (2005). PAHs in sediments of the Black River and the Ashtabula River, Ohio: Source apportionment by factor analysis. Water Res..

[38] Chen Y.Y., Jia R., Yang S.K. (2015). Distribution and source of polycyclic aromatic hydrocarbons (PAHs) in water dissolved phase, suspended particulate matter and sediment from Weihe River in Northwest, China. Int. J. Environ. Res. Public Health.

[39] Canadian Council of Ministers of the Environment Polycyclic Aromatic Hydrocarbons. Canadian Soil Quality Guide-Lines for Protection of Environmental and Human Health. Canadian Soil Quality Guidelines.

[40] Zhao L., Hou H., Shangguan Y.X., Cheng B., Xu Y.F., Zhao R.F., Zhang Y.L., Hua X.Z., Huo X.L., Zhao X.F. (2014). Occurrence, sources, and potential human health risks of polycyclic aromatic hydrocarbons in agricultural soils of the coal production area surrounding Xinzhou, China. Ecotoxicol. Environ. Saf..

[41] Li G.L., Lang Y.H., Yang W., Peng P., Wang X.M. (2014). Source contributions of PAHs and toxicity in reed wetland soils of Liaohe estuary using a CMB-TEQ method. Sci. Total Environ..

[42] Yuan H.M., Li T.G., Ding X.G., Zhao G.M., Ye S.Y. (2014). Distribution, sources and potential toxicological significance of polycyclic aromatic hydrocarbons (PAHs) in surface soils of the Yellow River Delta, China. Mar. Pollut. Bull..

[43] Wang L.J., Zhang S.W., Wang L., Zhang W.J., Shi X.M., Lu X.W., Li X.P., Li X.Y. (2018). Concentration and Risk Evaluation of Polycyclic Aromatic Hydrocarbons in Urban Soil in the Typical Semi-Arid City of Xi’an in Northwest China. Int. J. Environ. Res. Public Health.

